# TM4SF10 gene sequencing in XLMR patients identifies common polymorphisms but no disease-associated mutation

**DOI:** 10.1186/1471-2350-5-22

**Published:** 2004-09-02

**Authors:** Christiane Christophe-Hobertus, Frank Kooy, Jozef Gecz, Marc J Abramowicz, Elke Holinski-Feder, Charles Schwartz, Daniel Christophe

**Affiliations:** 1 Institut de Recherche Interdisciplinaire en Biologie Humaine et Moléculaire (IRIBHM), Université Libre de Bruxelles, IBMM, B-6041 Gosselies, Belgium; 2 Department of Medical Genetics, University of Antwerp, Antwerp, Belgium; 3 Department of Genetic Medicine, Women's and Children's Hospital, Adelaide, Australia; 4 Department of Pediatrics, The University of Adelaide, Australia; 5 Service de Génétique Médicale, Hôpital Erasme, B-1070 Bruxelles, Belgium; 6 Medizinisch Genetisches Zentrum, Bayerstrasse 53, D-80335, Munchen, Germany; 7 Center for Molecular Studies, J.C. Self Research Institute, Greenwood, S.C. 29646, USA

## Abstract

**Background:**

The TM4SF10 gene encodes a putative four-transmembrane domains protein of unknown function termed Brain Cell Membrane Protein 1 (BCMP1), and is abundantly expressed in the brain. This gene is located on the short arm of human chromosome X at p21.1. The hypothesis that mutations in the TM4SF10 gene are associated with impaired brain function was investigated by sequencing the gene in individuals with hereditary X-linked mental retardation (XLMR).

**Methods:**

The coding region (543 bp) of TM4SF10, including intronic junctions, and the long 3' untranslated region (3 233 bp), that has been conserved during evolution, were sequenced in 16 male XLMR patients from 14 unrelated families with definite, or suggestive, linkage to the TM4SF10 gene locus, and in 5 normal males.

**Results:**

Five sequence changes were identified but none was found to be associated with the disease. Two of these changes correspond to previously known SNPs, while three other were novel SNPs in the TM4SF10 gene.

**Conclusion:**

We have investigated the majority of the known MRX families linked to the TM4SF10 gene region. In the absence of mutations detected, our study indicates that alterations of TM4SF10 are not a frequent cause of XLMR.

## Background

Brain Cell Membrane Protein 1 (BCMP1) cDNA was fortuitously isolated from a thyroid cDNA library [1]. It encodes a 181aa-long putative four-transmembrane domain protein which appears related to both Peripheral Myelin Protein 22 / Epithelial Membrane Proteins and Claudins protein families, and exhibits significant similarities to the *Caenorhabditis elegans *VAB-9 protein, a protein that has recently been shown to be involved in the control of cell adhesion and epidermal morphology [2]. The protein sequence itself has been extremely well conserved during evolution, as exemplified by the observation that human and canine sequences are identical and differ from the mouse sequence at only 2 positions. The corresponding gene has now been renamed TM4SF10 in man and mouse, and is located on the X chromosome in both species, as well as in the other mammalian species investigated to date [1].

Initial Northern blot analysis of TM4SF10/BCMP1 gene transcripts distribution in adult dog tissues revealed very high expression in the brain, and lower but clearly detectable levels of expression in most of the tissues examined [1]. Data mining in the SAGEmap database [3] confirmed this observation in man, as elevated tag counts have been reported in brain astrocytoma (SAGE H127 library), brain ependymoma (SAGE ependymoma 353 and 582 libraries) and normal spinal cord (SAGE normal spinal cord library) as compared to other tissues. Together with its localization on the X chromosome, the high expression level detected in the brain and the putative role of the encoded protein in specific cell contacts raised the possibility that the TM4SF10 gene may be involved in X-linked mental retardation (XLMR) in man.

Initially, the TM4SF10 gene was assigned to Xp11.4 [1]. As the integration between human cytogenetic and DNA sequence-based maps is still evolving, the gene has been reassigned to band p21.1. It is noteworthy that TM4SF2, another gene encoding a four-transmembrane domain protein, is located at the p11.4-p21.1 border on human chromosome X, in the very close vicinity of TM4SF10, and constitutes a known XLMR gene [4,5]. Recent compilations of XLMR families [6-8] mention several conditions mapped to the Xp11.4-p21.1 region. We report the result of mutation screening of TM4SF10 in a cohort of XLMR patients whose gene was mapped to this region of the X chromosome and does not correspond to TM4SF2.

## Methods

Blood genomic DNA was collected from 16 patients (14 unrelated) and 5 unrelated healthy volunteers using a standard procedure [9]. The patients were affected males from families with definite, or possible, linkage to the region at Xp11.4-p21.1. Patients belonged to the following published MRX(S) families: MRX9 [10], MRX10 [11], MRX11 [11], MRX12 [11], MRX18 [12], MRX56 [6] and MRXS10 [13]. Additional patients were from an XLMR family with epilepsy [14] and 4 other XLMR families (C.S., F.K., J.G., unpublished), a MRXS family with macrocephaly and large ears (C.S., unpublished), and another MRXS family (J.G., unpublished). Chromosomal linkage data and major phenotypic traits are described in table 1. All samples were studied anonymously and all procedures met the standards of our institutional ethics committee.

**Table 1:** Description of the patients included in the study

Patient	Family	Linkage data	Phenotype
P1, P2	XLMR family (F.K., now MRX79)	chromosome X	X-linked mental retardation
P3, P4	MRXS10	Xp11.21-Xp11.4	Mental retardation, choreoathetosis, abnormal behavior
P5	XLMR family (F.K.)	Chromosome X, pericentromeric	Non-syndromic mental retardation
P6	MRX9	Xp11.22-Xp11.4	Non-syndromic mental retardation
P7	MRX10	Xp11.3-Xp21.2	Non-syndromic mental retardation
P8	MRX11	Xp11.3-Xp21.2	Non-syndromic mental retardation
P9	MRX12	Xp11.21-Xp21.2	Non-syndromic mental retardation
P10	MRX18	Xp11.3-Xp21.2	Non-syndromic mental retardation
P11	XLMR family with epilepsy	Xp11.23-Xp22.22	Non-syndromic mental retardation, epilepsy
P12	MRXS family (J.G.)	Xp21.3-Xq21.3	Non-syndromic mental retardation
P13	XLMR family with macrocephaly (J.G.)	Xp11.4-Xq13.1	Macrocephaly, moderate to profound mental retardation
P14	XLMR family (C.S.)	Xp11.3-Xp21.1	Seizures, ataxia, aggressive and hyperactive behavior, speech delay, mild to moderate mental retardation
P15	MRXS (C.S.)	Xp22.22-Xq24	Macrocephaly, prominent ears and moderate to severe mental retardation
P16	MRX56	Xp11.21-Xp21.1	Non-syndromic mental retardation

PCR reactions were performed in a final volume of 100 μl containing 200 ng of genomic DNA, 1 μg of each primer (see table 2 for primer sequences), 200 μM of each dNTP, 1X PCR buffer (QIAGEN) and 2.5 units of Taq polymerase (QIAGEN). Additionally, 10% DMSO or 20% Q solution (QIAGEN) were also included in some reactions (see table 2). After an initial denaturation step (93°C, 45 sec.), 35 cycles were conducted as follows: 93°C, 45 sec.; annealing temp. (see table 2), 45 sec.; 72°C, 45 sec. (amplicons Ex1–Ex3) or 1 min. (amplicons 3'UTR F1–F4). A final extension step (72°C, 3 min.) was done at the end of the reaction. PCR products were purified using the QIAquick PCR purification kit (QIAGEN) before sequencing. Purified PCR fragments were roughly quantitated by agarose gel electrophoresis using SmartLadder molecular weight marker (EUROGENTEC) as a quantitative reference.

**Table 2:** PCR conditions and primer sequences used to amplify TM4SF10 gene fragments from total genomic DNA

Amplicon	Size	Primer sequence	PCR conditions
Ex1	442 bp	fw: AGAGCCCCGAGGGAGCGA, rev: GGGGACAGGCGGTGACTG	T_anneal _= 55°C, 10% DMSO
Ex2	447 bp	fw: AAATCCTAGCAAACCCCTGG, rev: TCTGCATAGGAAAGGAAGATGG	T_anneal _= 50°C
Ex3	447 bp	fw: CCATCTAGAACAAGCCATCTTTAA, rev: TAAATCAACTGAGCAAACTGCTTG	T_anneal _= 50°C
3'UTR F1	959 bp	fw: GGCCTGGGGTGCAACTATAT, rev: TAGGCAAATGTATGTGGAGGGT	T_anneal _= 55°C, 10% DMSO
3'UTR F2	1101 bp	fw: ATTGGTGCCTCAGCCCTATCTA, rev: GCAACCATTCTTAAGACAAGCT	T_anneal _= 57°C, 20% Q solution
3'UTR F3	1130 bp	fw : CAGTATGTTCTGGTTTTGGCCC, rev : TATCTAACAATGGGTTTGTGGC	T_anneal _= 57°C, 20% Q solution
3'UTR F4	1097 bp	fw : CCTTCTCAGCAAAGAGCCCTAC, rev : AAGGATCTTGGGAGATAATTTG	T_anneal _= 57°C, 20% Q solution

About 50–100 ng of purified PCR fragment was used in a DNA sequencing reaction using a nested, internal primer. DNA sequencing was performed using ABI PRISM BigDye Terminator Cycle Sequencing Ready Reaction kit (Applied Biosystems) on an Applied Biosystems 3100 automatic DNA sequencer. The sequences of the primers are given in table 3.

**Table 3:** Sequences of the primers used for sequencing of TM4SF10 gene fragments

Amplicon	Primer sequence
Ex1	fw: CCGAGGGAGCGAGTCCCC
Ex2	fw: CACATCTGTTGAGCCACTGC
Ex3	rev: GATGCTCCACAAGTGTTTTAGA
3'UTR F1	fw : TGCCTGAACCCTAAGAACTATG, rev : GGAGGGTTAGGGAACAACTTAT
3'UTR F2	fw : CTGCATGAGTTGCTTTTGTACC, rev : GCAACCATTCTTAAGACAAGCT
3'UTR F3	fw : TCTGTTAAGAGCAGGACCACAT, rev1: ACTCGAGATGTGATGATATTGG, rev2 : TATCTAACAATGGGTTTGTGGC
3'UTR F4	fw : AACATGAAAATTGTTGCTTCTC, rev : AAGGATCTTGGGAGATAATTTG

## Results and discussion

### Sequencing of TM4SF10 coding region

**Figure 1:**
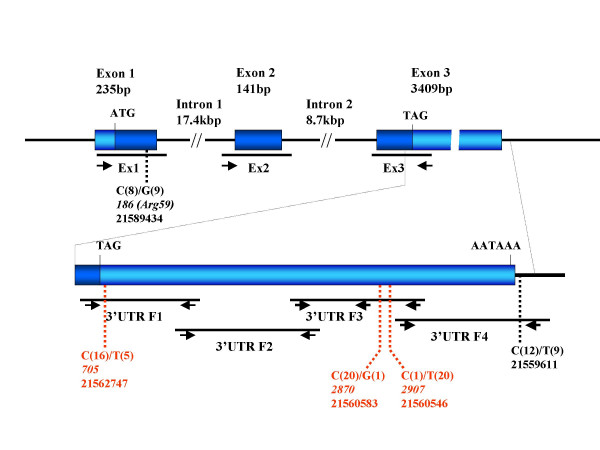
**Schematic of the TM4SF10 gene and single nucleotide polymorphisms identified in the study. **The structure of the gene is outlined with exons represented as light-blue boxes and the coding region as dark-blue areas within the boxes. The 3' end of the gene is also enlarged (bottom). Amplified regions are delineated and locations of sequence primers (arrows) used in this study are depicted. Identified SNPs are indicated (dotted lines) with reference to genomic contig (GenBank accession number NT_011757) sequence coordinates and cDNA (clone DKFZp761J17121; GenBank accession numberAL136550) sequence coordinates (italics) when relevant. Numbers in parentheses indicate the occurrences of the nucleotide in the individual sequences. The 3 novel SNPs identified in the work appear in red.

The human TM4SF10 gene is composed of 3 exons and produces a 4 kb-long mRNA. The short coding region (543 bp) is interrupted by 2 introns and the last and the largest exon also contains a 3 233 bp-long 3'UTR (see Figure 1). Initially, we sequenced the coding region and exon-intron junctions of the gene in the DNAs from 16 XLMR patients and from one normal male (amplicons Ex1–Ex3, Figure 1). No mutations were identified. One silent polymorphism was observed at position 186 in the cDNA sequence (clone DKFZp761J17121; GenBank accession number AL136550), corresponding to the 3^rd ^base of the codon Arg59, where a C residue was present in half of the sequences and a G residue in the other half. Individual single nucleotide polymorphism (SNP) data are shown in table 4. It is noteworthy that patients P3 and P4 who belong to the same family exhibit a difference in their TM4SF10 gene sequence at this level. This observation argues against a causal role of the gene in this family. The 186C>G polymorphism in the TM4SF10 gene had been previously reported [15].

**Table 4:** Description of the individual TM4SF10 gene SNP haplotypes determined in this study

	Exon 1: 186 (Arg59), [21589434]	3' UTR: 705, [21562747]	3' UTR: 2870, [21560583]	3' UTR: 2907, [21560546]	3' intergenic: [21559611]
N1	C	C	G	T	C
N2	(n.d.)	C	C	C	C
N3	(n.d.)	C	C	T	C
N4	(n.d.)	T	C	T	T
N5	(n.d.)	C	C	T	C
P1	C	T	C	T	T
P2	C	T	C	T	T
P3	G	C	C	T	C
P4	C	C	C	T	C
P5	G	C	C	T	T
P6	G	C	C	T	T
P7	G	C	C	T	C
P8	G	C	C	T	C
P9	G	C	C	T	C
P10	C	C	C	T	T
P11	C	T	C	T	T
P12	G	C	C	T	C
P13	G	C	C	T	C
P14	C	C	C	T	T
P15	C	T	C	T	T
P16	G	C	C	T	C

The first row identifies the source of the DNA, either a normal individual (Nx) or a patient (Px; see also table 1 for the detailed description of the patients). The individual bases found at each polymorphic position in the DNA sequence (identified by the nucleotide position in the cDNA sequence and/or in the genomic contig sequence [in square brackets]; see also figure 1) are given in the following rows. (n.d.): not determined.

### Sequencing of the 3'UTR

The long 3'UTR sequence of the TM4SF10/BCMP1 transcript is highly conserved, with an overall score of 72% when human, dog, mouse and rat sequences are compared. As the 3'UTR of mRNAs may contain regulatory sequences that participate in the control of gene expression, we decided to screen this part of the gene as well. The entire region, including the sequences around the polyadenylation site, was subdivided into four overlapping fragments of approximately 1 kb in length (3'UTR F1–F4, Figure 1) and sequenced from both ends. In fragment 3' UTR F3 the presence of a stretch of 12 consecutive A residues on the sense strand resulted in difficulties in proper reading of the sequences located downstream of this motif. In order to overcome this problem, an additional sequence primer (rev2) was used to obtain overlaps between the 3 separate sequences for each individual fragment. In the cDNA sequence AL136550 the motif is composed of 13 A residues, which is a likely sequence artefact.

TM4SF10 sequence was obtained from 16 patients and 5 controls. Four SNPs were identified in the non-coding part of the gene: 3 of them were located in the 3'UTR of the mRNA while the fourth one was located downstream of the polyadenylation site (Figure 1). Only this last one (C21559611T) had been previously reported in the SNP database [15], the other three representing novel SNPs in the TM4SF10 gene. The individual SNP haplotypes determined here are described in table 4. It is also noteworthy that during the course of our investigation, the genetic defect of one of the unpublished XLMR family included in the study (see top of table 1) has been identified and mapped to Xq28, within the MECP2 gene [16].

## Conclusions

In this study, we have investigated the majority of the known MRX families linked to the TM4SF10 gene region. In the absence of mutations detected, our results indicate that alterations in the transcribed region of TM4SF10 are not a frequent cause of XLMR. Although the gene promoter has not been identified and screened yet, it appears very unlikely that all mutations would be there.

This work also identified three novel SNPs in the TM4SF10 gene, which adds to our knowledge of SNP occurrence within this gene.

## Competing interests

None declared.

## Authors' contributions

CCH performed/managed the PCR and sequencing reactions, and analyzed the DNA sequences. FK, JG, MJA, EHF and CS provided the DNA samples. These authors also participated in the writing of the manuscript. DC conceived and supervised the study, and drafted the manuscript. All authors read and approved the final manuscript.

## Pre-publication history

The pre-publication history for this paper can be accessed here:


